# What Really Matters to Survivors of Acute Type A Aortic Dissection—A Survey of Patient-Reported Outcomes in the Dutch National Aortic Dissection Advocacy Group

**DOI:** 10.3390/jcm12206584

**Published:** 2023-10-18

**Authors:** Jennifer S. Breel, Eline S. de Klerk, Magnus Strypet, Frederiek de Heer, Henning Hermanns, Markus W. Hollmann, Susanne Eberl

**Affiliations:** 1 Department of Anesthesiology, Amsterdam University Medical Centers, Location Meibergdreef, 1105 AZ Amsterdam, The Netherlands; j.s.breel@amsterdamumc.nl (J.S.B.); e.s.deklerk@amsterdamumc.nl (E.S.d.K.); m.strypet@amsterdamumc.nl (M.S.); h.hermanns@amsterdamumc.nl (H.H.); s.eberl@amsterdamumc.nl (S.E.); 2 Department of Cardiac Surgery, Amsterdam University Medical Centers, Location Meibergdreef, 1105 AZ Amsterdam, The Netherlands; f.deheer@amsterdamumc.nl

**Keywords:** cardiac surgery, health related quality of life, communication, expectations, postoperative outcomes

## Abstract

(1) Background: Type A acute aortic dissection (TAAAD) almost always requires emergency surgery, and postoperative complications are common. Quality assurance systems tend to measure only the hard outcomes, e.g., complications and mortality. Our aim was to assess the health-related quality of life of TAAAD survivors. (2) Methods: An anonymized, Dutch language, web-based survey was sent out to all of the participants of the 2022 Annual Meeting of the Dutch National Aortic Dissection (DNAD) advocacy group via their own representatives. The survey was divided into five sections: patient information, global satisfaction, surgery, including complications, and the recovery period. (3) Results: Ninety members of the DNAD group attended the meeting. Seventy-five (83%) participated in the survey, and the responses from 61 (81%) were available for analysis. Despite hindrances in their daily life (complications, changes in physical, cognitive, and social functioning), patients were satisfied with their treatment, and all would undergo the procedure again. In addition they requested better post-discharge guidance and communication (4) Conclusion: The emphasis lies in equipping patients with knowledge about potential outcomes and effective coping strategies. This underscores the importance of communication and expectation management, in line with established literature.

## 1. Introduction

Type A acute aortic dissection (TAAAD) is a rare but potentially lethal event, with an incidence of approximately 3/100,000 people. Diagnosis is usually made using the European Society of Cardiology guideline and algorithm [1]. In addition, guidelines for the role of multimodal imaging are used in facilitating a fast and accurate diagnosis [2]. Emergency surgery is almost always necessary, and postoperative complications are common. It is important to understand that not only early complications, such as mortality and short-term morbidity (<30 days), need to be measured, but also a wide range of adverse events with long-term effects (such as stroke, renal failure, heart failure, cognitive dysfunction, etc.), as these also have a serious impact on the quality of life of surviving patients [3]. Most of the aforementioned complications are hard outcomes [4], collected in quality assurance registers, to measure or benchmark surgical success. However, surgical success and patient satisfaction with the Health Related Quality of Life (HRQoL) are two very different sides of the story.

It has become evident in recent years that the measurement of surgical outcomes is often one-sided, i.e., without the input of the patients themselves [5]. Patient-Reported Outcomes (PROs) are essential to evaluate the influence of a medical intervention on HRQoL. PROs are self-reported measures of a patient’s actual health status, measured using PROMs (Patient-Reported Outcome Measures) and Patient-Reported Experience Measures (PREMs). PROs reflect the patient’s views and perceptions, as well as experiences with healthcare from a personal perspective [6,7].

PROs are standardized tools that should at least measure the four main domains: global, mental, physical, and social health. They are either generic to multiple diseases/procedures or specific to one [8]. Examples of a generic HRQoL that are commonly reported in literature are the EuroQol (EQ-5D-5L) [9], the Short-Form Health Survey (SF-36 or SF-12) [10,11] or the WHODAS 2.0 score [12], the Hospital Depression and Anxiety Questionnaire (HADS) [8,13], and the Patient-Reported Outcomes Measurement Information System (PROMIS) [14].

A challenging aspect in the measurement of HRQoL is that the generic questionnaires are designed to capture a wide range of populations and thus may not capture specific concerns; they have a limited depth of information, they may not capture complex or multi-faceted outcomes, show a potential for response bias, and, due to non-standardized scoring systems, make comparisons across studies difficult. In addition, they may not be sensitive enough to detect small or specific changes over time. For example, the EQ5D measures the HRQoL of the measurement day, whilst the SF-12 and -36 measure HRQoL over the last month.

In practical terms, this means that it is difficult to assess patients cross-sectionally with the same disease, but from different time-periods since the operation. The baseline for such a measurement would differ too widely to draw accurate conclusions. Another bias in patient outcome surveys is that patients often have very different levels of information about their disease, the operation, the postoperative course, and the expected long-term effects. If patients are not directly involved in the selection of outcomes that are meaningful to them, it is possible that a mismatch can occur with what clinicians find important [5].

We aimed at overcoming these limitations and obtaining more truly patient-centered information, including which outcomes are important to patients and suggestions for improving care and decision-making in the population of aortic dissection patients in the Netherlands. To obtain this information, we performed a survey amongst the participants of the 2022 Annual Meeting of the Dutch National Aortic Dissection (DNAD) advocacy group. The DNAD consists of people who have survived aortic dissection and have joined together to support, educate, and advocate for others dealing with the same disease.

The content of the survey was to ascertain which patient-centered outcomes were important in five domains: patient characteristics, patient satisfaction, surgery-related questions, including complications, and the recovery period.

## 2. Materials and Methods

### 2.1. Ethics

The Medical Ethics Review Committee of the Amsterdam UMC confirmed that the Medical Research Involving Human Subjects did not apply to this survey (10 February 2022, W22_051#22.082). We adhered to the principles of the Declaration of Helsinki (Fortaleza), Good Clinical Practice, and the General Data protection Regulation (GDPR). The survey was developed using the Consensus-Based Checklist for Reporting of Survey Studies (CROSS) guideline [15].

### 2.2. Participants

All adult members of the DNAD (*n* = 300) who had survived an acute aortic dissection event, regardless of when it had occurred, were eligible. All participants (*n =* 90) of the 2022 Annual Meeting of the Dutch National Aortic Dissection (DNAD) advocacy group on 18 September 2022 at the Catharina hospital, Eindhoven, the Netherlands, were invited to complete the survey.

### 2.3. Survey

The Dutch language, web-based, prospective, open survey was created in Survey Monkey (Palo Alto, CA, USA) and consisted of a total of 27 questions, in the following domains: patient demographics (sex, year of birth, height, weight, previous medical history, educational level, marital status), patient satisfaction (about information, treatment received, global satisfaction), surgery (including postoperative complications), and the recovery period (physical ability, employment status, change in home environment, and open questions regarding information that the patient would like to have received and points to improve on). The majority of questions were multiple choice where only one answer was possible. A number of the additional questions were open text, where patients could express their opinions [see supplementary material (Supplementary Table S1) for an English translation of the questionnaire]. A Metabolic Equivalent of Task score was used to assess physical functioning, where a higher score means a higher level of functioning. Patients were asked to recall their functioning before the operation in comparison to that after the operation.

The survey was developed by two cardiac anesthetists, a clinical epidemiologist, and a research coordinator. All of these persons have a background in research into and with this patient group. After the initial draft, it was revised several times by the authors in order to produce the most suitable questions and to determine the wording and sequence of the format. It was tested on members of the DNAD committee for additional expert input for the final version.

Thereafter, a short description of the aim of the survey, together with the link to Survey Monkey, was sent to the DNAD Committee, who e-mailed it to the members attending the Annual meeting on 18 September 2022 in the Catharina Hospital in Eindhoven, the Netherlands.

In the e-mail accompanying the link, members were informed that participation was voluntary, that the survey was anonymous, and that if they took part, they gave permission to analyze and use their data. The survey link was active from 18 August 2022 to 17 September 2022. One reminder was sent after two weeks by the DNAD. The survey was de-identified; no direct link could be made to the patients. The data were then transferred to a Castor EDC database for analysis.

Survey data were handled by three members of the team, who analyzed and wrote the manuscript.

### 2.4. Sample Size

No formal sample size calculations were made as there was no formal hypothesis and no validity or reliability testing was planned. The survey was intended to be purely descriptive in nature. We hoped to receive an answer from at least 45 (50%) of the persons to whom a link was sent. We accepted a missing data response of <5%. Missing data were considered missing not at random (for example the year of birth). No data were imputed.

### 2.5. Statistics

The results are presented as frequencies with percentages, means with standard deviations, or medians with inter-quartile ranges. Data are presented for the entire cohort of respondents, divided by sex.

## 3. Results

### 3.1. Basic Survey Results

**Figure 1:**
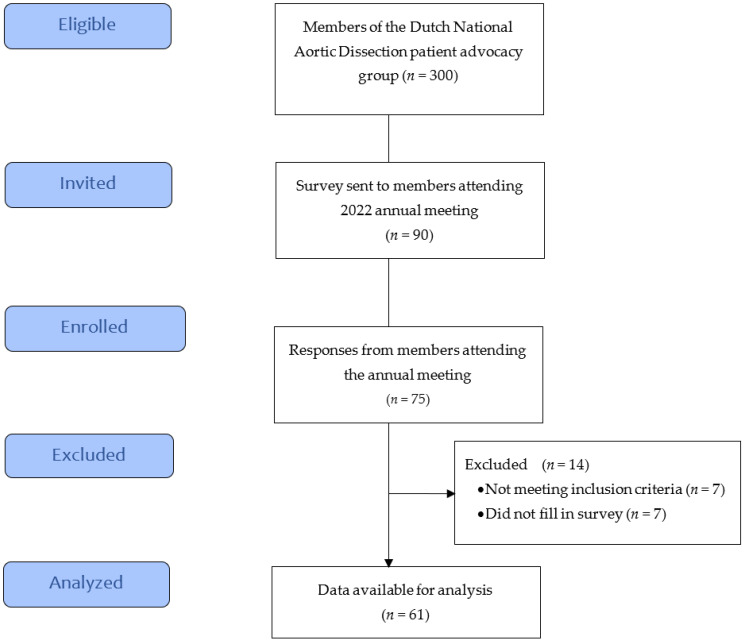
Flowchart of the TAAAD survey.

The response rate of the survey was 83%. Of the 90 patients contacted, 75 completed the questionnaire; seven patients were excluded because they were treated conservatively for a type B dissection, in addition to seven who started the survey but did not fill in any data. For the final analysis, 61 patients who had undergone surgery for type A dissection were included. (Figure 1). Eleven patients (18%) underwent surgery within the past year, 30 (49%) one to five years ago, and 20 (33%) more than five years ago.

### 3.2. Patient Characteristics

#### 3.2.1. Basic Patient Information and Medical History

Patient characteristics are shown in Table 1. The mean age was 62 (±10) years. Nineteen (31%) were female. The mean height was 178 (±8) cm and the mean BMI was 27 (±4) kg/m^2^. The majority (59%) reported no relevant personal medical history; however, 34 (56%) had a positive family history of heart disease.

#### 3.2.2. Social Background

Most of the patients were married or living in a partnership (44 (72%)). Forty patients (66%) were still working. Educational levels showed that twelve (20%) had completed high school, 34 (56%) had completed higher education, and nine (15%) were educated at the university level.

### 3.3. Patient Satisfaction

Fifty-one (84%) patients were satisfied or very satisfied with the care they had received at the hospital, although 28 (46%) stated that they were not very well informed before surgery. However, in open responses, they added that this was due to the emergency nature of the operation and that their relatives were very well enlightened. Almost half of the patients (31 (51%)) reported that their recovery was different to what they had expected, especially in terms of mental recovery. Nevertheless, in the response to the question “Would you make the same decision (i.e., to have surgery) again with the knowledge you have now?”, 46 (75%) answered “yes”. Fourteen (23%) indicated that they had had no control over this decision (due to unconsciousness). Importantly, none of the patients reported that they would not undergo surgery again despite complications.

### 3.4. Surgery including Complications

As expected, 61 (100%) were emergency procedures. Only 30 (49%) had an acute pain event a few hours before surgery. Five (8%) had an acute pain event days before the dissection, and three (5%) had it weeks before. Of note, eight (13%) patients had no symptoms at all before dissection.

Complications (more than one answer possible) were reported by 25 (41%) of all patients after surgery. Of the major complications, nine (15%) reported new cardiac complaints, seven (12%) cerebral, one (2%) renal problems, and seven (12%) reported chronic pain disorders. Mild complaints, such as various cramps, dizziness, or concentration problems were seen in 24 (39%) of the patients.

Importantly, eight (13%) were hospitalized once and 16 (26%) more than once in association with late sequelae of dissection. The other 32 (52%) were not readmitted.

### 3.5. Recovery Period

#### 3.5.1. Physical Functioning

Questions on physical performance were based on an adjusted MET score; patients compared physical functioning pre- and post-procedures. It was found that for a MET score of 1–3 (self-sufficient health care, slow walking), 36 (59%) reported having reached their pre-surgery state. A MET score of 4–6 (light housework, letting the dog out, walking slowly) was achieved by 25 (41%). Faster cycling or swimming (MET 7–9) was something only seven (12%) could carry out at the pre-surgery level; 52 (81%) were worse than before surgery or unable to reach this level of energy expenditure.

#### 3.5.2. Cognitive Functioning

When asked about cognitive functioning, 31 (51%) patients reported worse cognitive functioning. Twenty-five (41%) could still read, write, or speak in social situations for longer than ten minutes, at the same level as before surgery. In contrast, 33 (54%) reported worse, significantly worse, or being unable to function for ten minutes, while 30 (49%) reported cognitive issues, such as memory loss and a concentration span reduced to less than ten minutes. Persistent fatigue was reported in 24 (39%).

Overall, this general cognitive deterioration was not associated with more depression or anxiety than prior to surgery. Sixteen (26%) and eleven (18%) patients, respectively, still suffered from anxiety and depression at the same level as that previously, two (3%) reported more anxiety, and three (5%) more depressive episodes than before surgery.

Fifteen patients (25%) reported suffering from both anxiety and depression; five (8%) suffered only from anxiety.

#### 3.5.3. Social Functioning

Fifty-two (85%) patients reported that their living situation was the same as that before the operation. Fourteen (23%) required daily medical care and ten (16%) support with household chores, which was not the case before. This was also reflected in their professional life. Only thirteen (21%) were able to resume their previous employment. In addition, 33 (54%) of all those affected would have liked more physical and mental support.

#### 3.5.4. Further Needs

In the open-text fields, patients complained of a lack of communication, gaps in memory, slow recovery with extreme fatigue, complications with sequelae, restrictions in ADL, and the lack of good guidelines for postoperative care. One of the most important “open question” points for patients was to receive more information about the post-operative trajectory and also more support in this phase. They found it important to have a postoperative roadmap with a case manager, which indicates approximate recovery times, limits for physical progress, but also ranges for physiotherapy, and physical activity. Emotional and mental support was also a frequently mentioned point. Since an “aortic prosthesis” is a disease that is not “visible” from the outside, many patients felt that they were not taken seriously by their environment during their rehabilitation. Similar to all patients in the corona period, TAAAD patients experienced the limitations of having limited contact with family. Retrospectively, they often miss what happened during their first time in the ICU and would like to receive a “diary” with day-to-day details to “fill in” this period.

## 4. Discussion

We conducted a survey among survivors of a type A acute aortic dissection event to identify the most important patient-centered outcomes for this group. With a robust response rate of 83%, it is clear how valuable it was for the patients to actively contribute to a survey that examined their unique perspectives, expectations, and even suggestions for improvement.

### 4.1. Patient Postoperative Perceptions and Expectations

Patients reported that the occurrence of surgical complications had an enormous impact on their postoperative psychosocial outcomes and well-being. As reported by Pinto et al., these complications can have long-term effects and may result in challenges that affect patients’ recovery and overall well-being [16].

The key focal point, however, was the substantial gap between patients’ expectations for postoperative recovery and the tangible outcomes they faced. Patients reported that the postoperative recovery process was not what they had expected. This was despite the fact that the discrepancies between health care issues and reality have been thoroughly researched and are well known to health-care providers. The primary issue, therefore, appears to be a lack of effective communication and providing information to the patient about these aspects. The central concern of many survey patients was an unexpected decrease in cognitive ability, physical functioning (measured based on activities of daily life), and HRQol.

#### 4.1.1. Cognitive Changes

One of the most significant challenges involved a shift in energy levels and vitality: patients indicated experiencing fatigue and difficulties with concentration. Out of the surveyed patients, almost 40% indicated experiencing persistent fatigue, while almost half reported cognitive issues, such as memory loss and their concentration span being reduced to less than ten minutes. Patients also described aphasia, forgetfulness, lack of physical and mental stamina, inability to deal with stimuli, anxiety, and depression, as well as uncertainty with regard to the future

These cognitive disturbances persisted beyond five years after the event, observable in 33% of the patients surveyed. These outcomes are comparable with the investigations conducted by Rovai et al. and Bruggemans, who reported similar incidences ranging from 33% to 40% [17,18].

Before the dissection event, eleven (18%) patients were known to suffer from depression or anxiety (16 (26%)). However, after surgery, only a small percentage of patients reported an increase in anxiety or depressive episodes (two and three patients, respectively). It is well documented that the emotional toll of a TAAAD, especially the prolonged recovery and long-lasting disability, can increase the incidence of anxiety, depression, and even post-traumatic stress disorder significantly in survivors of TAAAD [16]. In the systematic review by Pinto et al., two-thirds of the included studies found a significant negative association between the occurrence of surgical complications and patients’ postoperative well-being [16].

Post-traumatic stress disorder (PTSD) is also often seen postoperatively after cardiac surgery. In particular, patients who require urgent surgery may be at risk.

Rovai et al. described that up to 18% of patients may experience a form of post-traumatic stress disorder (PTSD) after cardiac surgery [18]. Chen et al. also examined the incidence of PTSD after acute dissection [19]. They showed that at-risk patients had a higher prevalence of depression and anxiety. In addition, they reported a difference in risk factors affecting males and females: a high HADS was associated with developing PTSD, whilst factors, such as a good education (university level), being strongly optimistic, and having a good support system, were seen as protective. Our study could not corroborate these findings, possibly due to the small sample size.

#### 4.1.2. Physical Functioning

In line with the literature, our patients reported changes in daily life after aortic surgery [3]. There was a significant decline in physical fitness, with half of the respondents reporting a worsened state than that prior to surgery. With a MET score below four, many patients felt their ability to live and function independently of household or personal care was severely impacted.

About a quarter of the patients required extra care from family or professional organizations. This change in the Activities of Daily Life (ADL) is reported frequently in the literature. De Heer et al. reported that soon after surgery, the impact of impairment is most noticeable. Thereafter, a degree of improvement is seen, and at a certain point, the patient stabilizes. Age plays a role in this process as the influence of time (and age) is detrimental to HRQoL [20]. The loss of independence in activities of daily living add to the psychosocial burden that patients experience and is a co-factor in social isolation and an increase in depression [18]. This is supported by the fact that after the operation, only half of the patients were still doing the same work than before, and approximately half were no longer able to work and were on disability grants or retired early.

#### 4.1.3. Patient Satisfaction and Measurement Tools

The most important message, however, was that most patients were satisfied with the outcome and the treatment in the institute where they underwent surgery (87%).

This is evident from the fact that not a single patient reported that they would refuse surgery if armed with current knowledge and faced with the same decision again. This is true despite the fact that patients faced many post-operative challenges, such as re-operations, complications, and a decrease in physical and/or mental abilities.

Admittedly, as the alternative to this surgery is, for the majority, death, this may be a biased question.

An additional challenge related to patient satisfaction is the measuring and quantifying of it, using available questionnaires. This is true for our patients, as well as reports in the literature [20].

Generic PROMs, such as the EQ5D and SF-36 or SF-12, WHODAS 2.0, and HADS, capture some aspects of physical, mental, and social functioning, but are further limited [14,20,21,22,23,24]. Although much better than only (single) objective outcome clinical parameters, such as mortality and/or morbidity or pain [4,6], these questionnaires do not cover the gamut of changes and the time period of the rehabilitation trajectory.

In addition, the scales used sometimes produce floor or ceiling effects. This signifies not recognizing when patients are operating below the lowest level or unable to advance to a higher level. Moreover, they do not address satisfaction or some of the most common complaints, such as fatigue, memory loss, and a lack of concentration.

Cardiothoracic surgery studies and surveys—using the above-mentioned questionnaires—often do not use the endpoints/questions that are most meaningful to patients in their daily lives [3]. Unfortunately, the correlation between the lowest complication rates and the highest HRQoL are not always consistent [12].

In the last years, the concept of the minimal clinically important difference (MCID) is gaining popularity. MCID is the smallest change that a patient finds important and which would result in a change in treatment [7,25]. Gradually, for many of the validated questionnaires, MCIDs have been calculated. These MCIDs are especially important in the context of the theoretical model of “survivorship” [21].

Patients who have undergone an acute aortic dissection event describe themselves as “survivors”. After their hospitalization, they move into a chronic phase of the disease, where they need to make significant changes in lifestyle and adapt to new “unfamiliarities” and uncertainties [21], where the smallest changes can make a difference. Based on this model, several studies argue—in line with our results—that conventional PROs, PROMs, and PREMs do not cover this patient group and that specific ones should be developed [7,21,22,23]. In addition, although not perfect, a score, such as the MCID or its equivalent, should be incorporated into the measurement process [25].

##### Limitations and Strengths

Patient selection for this survey could be rated as a bias and therefore a limitation. The patients, who were asked to participate were survivors of a type A acute aortic dissection, members of the DNAD patient advocacy group, and also attending the annual meeting of the group. We thus selected surviving patients who are informed more than average about their disease and treatment. In addition, we have introduced recall bias, as patients were asked to recall information about their lives now in comparison to after the operation. As this was more than a year for the majority, it is possible that some details are incorrect. The survey is not validated and the questions are therefore open to different interpretations by the patients. In attempt to mitigate this, we attempted standardization using Likert-type scales to measure the multiple choice questions and used a standardized score for physical functioning (MET score). In addition, we asked for suggestions they might have for improvements to the survey.

On the other hand, the baseline characteristics of the patients who participated in this survey matched those described in the literature for patients with acute type A aortic dissection event: a lower percentage of females patients, who were smaller, lighter, and in terms of educational background, had lower levels of schooling compared to males. The other baseline characteristics, type and frequency of complications postoperatively, were consistent with the literature [3,16]. We can therefore assume that they are representative of aortic dissection patients, making the survey generalizable to this population. In addition, although the sample size was relatively small, the response rate was very high.

### 4.2. Suggestions for Improvement in Postoperative Care

From the open fields in the survey, we collected data on patients’ suggestions for improvement in postoperative care.

Enhancing information-sharing and communication with our patients emerged as the primary recommendations. The information provided before emergency surgery is usually confined to the immediate procedure and the initial one to two days after surgery. This typically involves topics, like the surgical duration, admission to intensive care, mortality, and significant complications. It is also a well-known fact that in stressful situations, the person involved only retains a small amount of the information given, and what they remember is often incorrect [16]. In this survey, most of the patients responded that the communication with themselves prior to surgery was not optimal. Nevertheless, they were satisfied, since their relatives were given adequate explanations of the procedure and risks.

Patients found that it was more important to receive information for themselves (or their families) regarding the risks of the operation, the post-operative trajectory, expectations—especially for the long-term—and what kind of support is required [5]. It was therefore not surprising that 60% of patients in the open survey requested more emotional and mental care in the postoperative period. This certainly presents an opportunity for healthcare improvements, warranting additional research to identify the factors involved and the most effective methods of realization.

Consistent with our findings, existing literature reveals that patients often experience memory gaps resulting from the operation and admission process. They found it disturbing that they had “lost” time and could not recall days or events that had taken place. A general suggestion was that a daily “diary” or logbook of events be kept by medical staff and relatives to help fill in the gaps. In a systematic review regarding the use of diaries in the intensive care, Barreto et al. found that the use of a diary was associated with a reduction in the risk of depression and an improvement in the quality of life [26]. This may be a useful tool in improving both emotional and psychological well-being.

Most suggestions received in the survey, however, were directed to the longer-term postoperative trajectory. The main concern voiced was the abundance of uncertainties, leaving patients uncertain about where to access information to cope with the shifts and assist them in accepting the changes in their lives

The foremost recommendation, however, revolves around the effective management of patient expectations. It is essential to provide patients with enough information so that they can set goals to work towards in the recovery phase. These goals need to be evaluated and updated regularly to ensure that the patient achieves the maximum possible HRQoL. Due to the cognitive disturbances, a strategy of verbal information, backed up with written and visual information, may be the best way to manage goals and expectations.

Some of the abovementioned expectation management was tested in the adaptation and validation of a PREM for cardiac patients, the Cardiac Surgery Patient Expectations Questionnaire (C-SPEQ) [22]. They found that negative expectations were detrimental to recovery and quality of life. Improvement was seen in non-clinical aspects of patient outcomes. Their main recommendation was to improve pre-surgical expectations to provide optimal preparation, reduce negative expectations, and improve psychosocial outcomes [22]. This is of course difficult in emergency surgery, but improving patient expectations for postoperative recovery will aid in improving satisfaction and coping.

### 4.3. Recommendations

To enhance postoperative support, a multifaceted approach is advised. This could involve pairing new patients with experienced ones through a buddy support system, similar to approaches in cancer, COVID-19, education, workplace, and abuse groups. Furthermore, group coaching facilitated by patient advocacy groups, along with individual psychological assistance, is to be recommended. Additionally, a telephone app with information per time period on what to expect and where to get help or answers may also be beneficial. Lastly, as in other disciplines, using specialized nurses or Physician Assistants to monitor the rehabilitation and perform expectation management on a regular basis would provide patients with a stable source of reliable information [27].

### 4.4. Future Directions and Research Studies

The pilot study questions will be evaluated for suitability and the questionnaire adapted accordingly. Furthermore, some questions will be added for extra mental health screening (PTSD). The survey will then be sent out to all patients who have undergone a TAAAD operation in the Amsterdam UMC, since January 2021, and coupled with their clinical data. In addition to complications associated with cardiac surgery, such as stroke, renal failure, heart failure, and cognitive dysfunction, specific complications for TAAAD (tamponade, aortic valve regurgitation, and proximal or distal malperfusion syndromes) and their influence on HRQoL will be evaluated [28,29,30].

In addition, we envisage performing a similar survey on all patients who have undergone cardiac surgery in our institute in the same time period as mentioned above.

We are currently evaluating the current support systems and, together with the patients, are planning a postoperative trajectory that better suits patient needs.

## 5. Conclusions

In conclusion, the emphasis lies not in predicting specific problems, but rather in equipping patients with knowledge about potential outcomes and effective coping strategies. This underscores the importance of communication and expectation management, in line with the established literature. It is time to prioritize patient-centered well-being. We need to change the island mentality of “that’s not my department’” to one of holistic patient care to achieve the goals that the patients themselves want.

## Figures and Tables

**Table 1:** Patient characteristics for the TAAAD Survey.

	Total *n =* 61	Female *n =* 19	Male *n =* 36
Age [years] mean (SD) *	62 (10)	61 (11)	63 (10)
Height [cm] mean (SD)	178 (8)	170 (6)	182 (6)
Weight [kg] mean (SD)	82 (15)	78 (17)	92(12)
BMI [kg/m^2^] mean (SD)	27 (4)	27 (5)	28 (4)
Smoker n (%) *	15 (27)	6 (10)	9 (15)
Diabetes Mellitus *	2 (3)	1	1
Family history CVD *	36 (71)	10	25
Education *			
High school	12 (22)	3 (5)	8 (13)
College	34 (62)	13 (21)	21 (34)
University	9 (16)	2 (3)	7 (11)
Marital status *			
Married/Registered partnership	44 (80)	15 (27)	28 (50)
Divorced	4 (7)	2 (4)	2 (4)
Widowed	2 (4)	1 (2)	1 (2)
Single	5 (9)	1 (2)	4 (7)

All variables are absolute values and percentages and means and standard deviations. The column Other’s indicates persons who preferred not to say which sex they were. * denotes variables with missing values: age, *n =* 52; smoking, *n =* 56; diabetes mellitus, *n =* 45; family history of CVD, *n =* 51; education, *n =* 55; marital status, *n =* 55. Abbreviations: TAAAD = type A acute aortic dissection, COPD = chronic obstructive pulmonary disease, CVD = cardiovascular disease.

## Data Availability

The authors have included all raw data pertaining to the survey in Supplementary Table S1: Survey answers. Open questions are available only in Dutch upon request.

## Supplementary Materials

The following supporting information can be downloaded at: https://www.mdpi.com/article/10.3390/jcm12206584/s1, Table S1: TAAAD Survey: questions and responses.
