# Supplementary material for: What Really Matters to Survivors of Acute Type A Aortic Dissection—A Survey of Patient-Reported Outcomes in the Dutch National Aortic Dissection Advocacy Group
Source: J Clin Med. 2023 Oct 18;12(20):6584. doi: 10.3390/jcm12206584 (PMC10607692; doi:10.3390/jcm12206584)
Supplement: Supplementary file 1 [file jcm-12-06584-s001.zip › jcm-2644800-supplementary.pdf]

Journal of
Clinical Medicine

MDPI

TAAAD Survey Questions and responses

Table S1. TAAAD Survey: questions and responses.

	Satisfaction	Number of responses
	The following questions concern your satisfaction with your treatment:	
1	Overall, how satisfied are you with your treatment at the hospital where you underwent your heart/vascular surgery?	
	Very dissatisfied	2
	Dissatisfied	2
	Neutral	6
	Satisfied	20
	Very satisfied	31
	Missing	0
2	Why did you choose to undergo surgery?	
	No choice (emergency operation)	61
	I was experiencing increasing symptoms and they were causing discomfort	0
	I was afraid	0
	Other	2
	Missing	0
3	If other, would you please explain: OPEN TEXT	OPEN TEXT.
4	Would you, with the knowledge you have now, make the same decision to undergo surgery again?	
	Yes	46
	No	0
	Not applicable	15
	Missing	0
5	If no, why would you not choose surgery again? OPEN TEXT.	OPEN TEXT.
	We would like to evaluate if the information you received was written at a so you could understand it. We intend to use this information to enhance future healthcare services. We welcome your suggestions on how we can improve this	
6	Was the process of the surgery explained to you in understandable language, by your doctor(s), nurse, or other medical staff? (including everything that happened before, during, and after the surgery)?	
	Yes	31
	No	28
	Missing	2
7	If no, what could have been explained better? Please provide your suggestions here. OPEN TEXT.	OPEN TEXT.
8	Were you admitted to the hospital for more than a week for your heart/vascular surgery?	
	Yes	57
	No	3
	Missing	1
9	Did your recovery go as you expected?	
	Yes	28
	No	31
	Missing	2
10	If no, what did not go as expected? OPEN TEXT.	OPEN TEXT.
	**Operation**	

	The following questions are about your heart/vascular surgery	
11	How long ago was your first heart/vascular surgery operation?	
	< 6 months	3
	6-12 months	8
	1-5 years	30
	> 5 years	1
	5-10 years	13
	>10 years	6
	Missing	0
12	What type of heart/vascular surgery did you undergo?	
	Major blood vessel (aorta) in the chest (Type A dissection)	49
	Major blood vessel (aorta) in the abdomen (Type B dissection or aneurysm	0
	Heart valves	1
	Coronary arteries	0
	Combination of above	4
	Other	7
	Missing	0
13	If other, please give details: OPEN TEXT	OPEN TEXT.
14	Have you undergone heart or vascular surgery again since your initial procedure?	
	Yes	43
	No	17
	Missing	1
15	How quickly was your heart/vascular surgery performed?	
	Emergency (within 12 hours)	61
	Urgent (within 1 week)	0
	Planned (within 4 weeks)	0
	Postponed	0
	Missing	0

Functioning after the operation

	The following questions are about your functioning after the heart/vascular surgery	
16	If you were independent before the heart/vascular surgery (meaning you didn't require assistance with household chores or daily care), has your situation changed now?	
	Yes	24
	No	33
	Not applicable	3
	Missing	1
17	Work situation before the surgery:	
	Employed	31
	Self-employed	9
	Disability grant	3
	Retired	17
	Never worked	1
	Missing	0
18	If you were working shortly before the heart/vascular surgery: was it possible to perform the same job after the surgery?	
	Yes	13
	No	22
	Disability grant	5
	Different job	1

	(Earlier) retirement	3
	Other	8
	Not applicable	9
19	If No, or Other could you please explain? OPEN TEXT	OPEN TEXT.
20	Is your living situation the same as before the surgery?	
	Yes	52
	No	9
21	If no, what has changed? OPEN TEXT	OPEN TEXT.
22	Do you now require home care (family/friends) or home nursing?	
	Yes	4
	No	46
	Missing	11
23	Did you require home care (family/friends) or home nursing before the operation?	
	Yes	4
	No	57
24	Would you have liked more physical or emotional support/guidance around the surgery?	
	Yes	33
	No	28
	Missing	0
25	If yes, could you please explain? OPEN TEXT	OPEN TEXT.
26	Did you develop new symptoms after the surgery (that were different from before the surgery)*?	
	Bowels (e.g. constipation, diarrhoea, cramps)	0
	Heart (e.g.chest pain, palpitations, shortness of breath)	9
	Brain (e.g.stroke, one-sided paralysis)	7
	Kidneys(e.g. fluid restriction, dialysis)	1
	Pain(e.g. anywhere in the body except chest pain)	7
	Not applicable	16
	Other	25
27	If other, could you please explain? OPEN TEXT	OPEN TEXT.
28	After the heart/vascular surgery, were you been readmitted to the hospital for more than 24 hours?	
	No	32
	Once	8
	More than once	16
	Missing	5
	We would like to compare your health/functioning before and after the surgery. Can you indicate which of the following activities you are able to do?	
29	METs 1-3: Showering independently, getting dressed, using the toilet, walking slowly	
	Much worse	2
	Worse	17
	No change	36
	Better	2
	Much better	0
	Not applicable	3
	Missing	1
30	METs 4-6: Performing household chores (such as vacuuming), mowing the lawn, walking the dog, walking at a moderate pace (5 km/hr)	
	Much worse	11
	Worse	19

	No change	25
	Better	1
	Much better	2
	Not applicable	2
	Missing	1
31	METs 7-9: Cycling vigorously, swimming intensely, playing tennis, running at a fast pace (9 km/hr)	
	Much worse	10
	Worse	17
	No change	7
	Better	1
	Much better	0
	Can't do this	22
	Not applicable	3
	Missing	1
32	METs 10-12: Playing soccer, running at a high speed (>10 km/hr), swimming vigorously (>3 km/hr), rowing vigorously	
	Much worse	9
	Worse	12
	No change	3
	Better	0
	Much better	0
	Can't do this	25
	Not applicable	11
	Missing	1
33	Concentrating/reading/writing/making connections/expressing yourself in words for longer than 10 minutes	
	Much worse	9
	Worse	22
	No change	24
	Better	1
	Much better	0
	Can't do this	2
	Not applicable	2
	Missing	1
34	If you had anxiety attacks before the surgery, how frequent are the attacks now?	
	Much worse	2
	Worse	0
	No change	16
	Better	1
	Much better	1
	Can't do this	1
	Not applicable	39
	Missing	1
35	If you had depression before the surgery, how are you feeling now?	
	Much worse	0
	Worse	3
	No change	11
	Better	1
	Much better	1
	Not applicable	41

	Missing	4
	**General questions**	
36	What is your year of birth? (converted to age)	56
	Missing	5
37	What was your weight before the operation in kg?	56
	Missing	5
38	How tall are you in cm?	56
	Missing	5
39	Do you have one or more of the following diseases*:	
	Congenital heart defect	1
	Congenital vascular anomaly (e.g., Marfan, Ehlers Danlos)	5
	Autoimmune disease of the intestine (e.g., Crohn's disease, ulcerative colitis)	1
	Autoimmune disease of the thyroid (e.g., Hashimoto's)	0
	Depression	2
	Known genetic heart disease (e.g., Brugada syndrome)	0
	Lung diseases (e.g., COPD, emphysema)	6
	Hypothyroidism or use of (levo)thyroxine	2
	Acquired heart damage (e.g., after an infection like acute rheumatic fever, endocarditis)	11
	Acquired vascular damage (e.g., aneurysm, dissection)	6
	Not applicable	24
	Missing	
40	If you have diabetes, which medication do you use?	
	Tablets	1
	Insulin	0
	Combination of tablets and insulin	0
	No medication	1
	Not applicable (I do not have diabetes)	43
	Missing	16
41	Do you have a family history of heart or vascular disease? For example, think of heart attacks, aneurysms (artery dilation), atherosclerosis, etc.*?	
	Yes	36
	No	15
	Missing	10
42	How long before the surgery/treatment did your (heart) symptoms start?	
	Hours	30
	Days	5
	Weeks	2
	Months	1
	Years	4
	I did not have any symptoms	8
	Not applicable	6
	Missing	5
43	Did you smoke in the year before your operation?	
	Yes	41
	No	15
	Missing	5
44	Did you experience unexplained shortness of breath during exertion or at rest before your heart/vascular surgery?	
	Yes	14

	No	40
	Missing	7
	**Social background**	
45	What is the highest level of education you have completed?	
	Primary School	0
	High School	12
	Vocational Education (MBO)	17
	Higher Education (HBO	17
	University	9
	None	0
	Missing	6
46	What is your marital status?	
	Married/registered partnership	42
	Living together	2
	Single	5
	Divorced	4
	Widowed	2
	Missing	6
47	What was your sex at birth?	
	Male	36
	Female	19
	Prefer not to say	6
	Missing	
48	We would like to know if there are areas for improvement, additional information, or other details about your surgery/diagnosis that you feel were missing, and what you would definitely share with other fellow patients. OPEN TEXT.	OPEN TEXT.
49	Do you have any questions or comments about the survey? Are there things you would like to have added/removed? We welcome your suggestions. OPEN TEXT.	OPEN TEXT.

*Some fields had multiple answers available.
